# The times they are a-changin’: a proposal on how brain flexibility goes beyond the obvious to include the concepts of “upward” and “downward” to neuroplasticity

**DOI:** 10.1038/s41380-022-01931-x

**Published:** 2022-12-27

**Authors:** Cassiano Ricardo Alves Faria Diniz, Ana Paula Crestani

**Affiliations:** 1 School of Medicine, Campus USP, Ribeirão Preto, SP Brazil; 2 Center for Neuroscience, University of California, Davis, CA USA

**Keywords:** Neuroscience, Physiology

## Abstract

Since the brain was found to be somehow flexible, plastic, researchers worldwide have been trying to comprehend its fundamentals to better understand the brain itself, make predictions, disentangle the neurobiology of brain diseases, and finally propose up-to-date treatments. Neuroplasticity is simple as a concept, but extremely complex when it comes to its mechanisms. This review aims to bring to light an aspect about neuroplasticity that is often not given enough attention as it should, the fact that the brain’s ability to change would include its ability to disconnect synapses. So, neuronal shrinkage, decrease in spine density or dendritic complexity should be included within the concept of neuroplasticity as part of its mechanisms, not as an impairment of it. To that end, we extensively describe a variety of studies involving topics such as neurodevelopment, aging, stress, memory and homeostatic plasticity to highlight how the weakening and disconnection of synapses organically permeate the brain in so many ways as a good practice of its intrinsic physiology. Therefore, we propose to break down neuroplasticity into two sub-concepts, “upward neuroplasticity” for changes related to synaptic construction and “downward neuroplasticity” for changes related to synaptic deconstruction. With these sub-concepts, neuroplasticity could be better understood from a bigger landscape as a vector in which both directions could be taken for the brain to flexibly adapt to certain demands. Such a paradigm shift would allow a better understanding of the concept of neuroplasticity to avoid any data interpretation bias, once it makes clear that there is no morality with regard to the organic and physiological changes that involve dynamic biological systems as seen in the brain.

## Introduction

The etymology of neuroplasticity breaks it down into two basic morphemes: neuro- plastic; with the “plastic” originally meaning “suitable for molding” as it comes first from the Greek term “plastikós” and later from the Latin “plasticus”. As a concept, neuroplasticity means the nervous system’s ability to reorganize its structure and functioning in response to some stimuli, either intrinsic or extrinsic [1]. Although simple in definition, the historical ground behind this concept relies entirely on the shoulders of magnificent and pioneering scientists. From a philosophical perspective, plasticity roots, as a reference to the nervous system, are still a matter of debate and have been tracked back to Willian James (1890), Santiago Ramón y Cajal (1894) and Demor (1896) in the late 1800s or Lugaro (1906) and Minea (1909) in the early 1900s [2–4]. However, the practical relevance of the neuroplasticity only began to be unveiled in the mid-20th century by Paul Bach-y-Rita who built the concept of sensory substitution by proposing that other brain areas may assume functions previously mediated by a lost neural tissue [5]. Similarly, based on Wilder Penfield works that showed a motor and sensory cortical representation of the body [6], Michael Merzenich noted that the adult brain is actually a dynamic structure, with the cortical body representations constantly shifting its boundaries [7]. Intriguing, Merzenich’s findings were initially criticized by David Hubel and Torsten Wiesel, two eminent neuroscientists at Johns Hopkins who had found a critical period for visual cortex plasticity to allow visual processing input to be replaced by the non-deprived eye at the expense of the deprived one [8, 9].

All the field advancement has not come without the controversy of the unknown and, not surprisingly, for so long the adult brain was considered hard-wired, incapable of any accommodation. However, after some breakthroughs, neuroplasticity is now well recognized as a fundamental and lifelong brain property. Although partially compromised, compared to early neurodevelopment, adult brain remodeling is still salient and fairly required for learning/experience- and internal milieu-based behavioral adjustments [10].

Bearing in mind a broader perspective of a flexible brain, we will go through studies related to early neurodevelopment, aging, stress, memory, and homeostatic plasticity to propose that brain flexibility goes beyond the obvious to include the concepts of “upward” and “downward” to neuroplasticity. Such sub-concepts aim to clarify how neuroplasticity is not only about building, but also about dismantling.

## Neuronal disconnection of a prolix brain as a natural consequence of the neurodevelopment

Humans are born with tens of billions of brain cells and a single neuron may contact as far as 15,000 other cells, moreover a 3 years old child’s brain has established nearly 1000 trillion synapses [11]. The immature nervous system has a fundamentally redundant neuro-circuitry, as evidenced by the cortical dendritic spine density exceeded in childhood by one and a half to threefold that of adult’s brain [12, 13]. Higher synaptic density of the cerebral cortex was also found in early postnatal development of monkeys [14, 15], rats [16] and kittens [17], compared to adult animals. Cajal in the late 19th had already noticed that the spine density of pyramidal neurons throughout early postnatal development is greater than in adulthood [18].

Overabundant synapses were proposed as an endeavor by the brain to generate a diversity of connections beyond genetic anchorage, as activity-induced stabilization selects from the overproduced synapses those to remain [19, 20]. Accordingly, multiple retinal inputs converge onto immature lateral geniculate nucleus in both mice and ferrets, most of which are eliminated to just a few be maintained into adulthood [21, 22]. It was then proposed that the fuzzy retinogeniculate connections from early development would support the polishing of geniculocortical connections in late development to sharpen thalamocortical topography and likely also fine tune the orientation of cortical receptive fields [22]. Neuromuscular junction (NMJ) has also been a highly successful model to study developmental synaptic pruning. Early in development, one muscle fiber stablishes weak connections with about ten overly intermingled motor nerve axons, most of which (except for one) are further eliminated through the next postnatal couple of weeks [23]. Interestingly, increasing divergence in synaptic strength was observed before one of the two axonal inputs was removed from the shared mice muscle fiber, as the survivor earned vigor by increasing its quantal content and the defeated one became gradually weaker up to full withdrawal within 1 to 2 days [24]. Indeed, axons broaden their territory in response to previously evacuated sites, since the “soon-to-be-eliminated axon” still takes over the NMJ after laser removal of the strongest input [25]. Similarly, such refinement is widely established throughout early neurodevelopment on several occasions as a matter of axonal disconnection, as it follows: thalamic connection with the layer IV cells of the visual cortex is disrupted [26]; preganglionic inputs are disconnected from submandibular ganglion cells [27]; and climbing fibers disassemble from cerebellar Purkinje cells [28, 29]. The hippocampus is also target of developmental remodeling as the projections of pyramidal cells from the hippocampus to the medial septum are transient in rats and abruptly withdraw after birth [30]. Furthermore, the mossy fibers (infrapyramidal bundle) carrying intra hippocampal axons from the dentate gyrus (DG) to CA3 dramatically retract between the third and fourth week after mice are born [31].

Interestingly, early neurodevelopmental refinement has also been noticed from non-mammalian species, such as in the auditory systems of chicks [32] and in the visual system of tadpoles [33]. Drosophila metamorphosis prompts also to the loss of the dendritic arbor and axonal branches after puparium formation [34, 35]. Which means that the brain’s basic genetic program responsible for the ability to trigger early development-based abundance of synapses is, at least partially, presumable as evolutionarily conserved in different classes of animals, including invertebrates such as insects. However, different cellular mechanism may still be involved in synaptic pruning under different circumstances [36]. Besides, according to most evidence, developmental synaptic pruning or input disconnection has been usually acknowledged to be complete around early phases, or even when young people reach puberty or late adolescence. Consistently, callosal axons in newborn monkeys outnumber those in adults, and about 70% of these axons disappear months after birth [37]. Still, while young mice had 73% of the spines in layer-5 pyramidal neurons (primary visual cortex) stable over one-month interval, with changes primarily related to spine removal, adult mice had 96% of the spines stable for longer than 13 months [38].

Therefore, throughout early development, nervous system maturation has the highly dynamic process of neuronal remodeling as its hallmark and synaptic pruning is ubiquitously part of this complex rearrangement [39]. So, an embryonic and transient template, generous in promiscuous synapses, gradually gives place to a keen adult pattern of activity-driven neuronal connectivity as the repertoire of possible circuit configurations is pruning-based refined [36]. More than a passive process, developmental pruning follows Hebb’s rule whereas asynchrony, but no synchrony, between action potential achieves competing terminals to trigger input concurrence and further synaptic elimination [40].

**Fig. 1:**
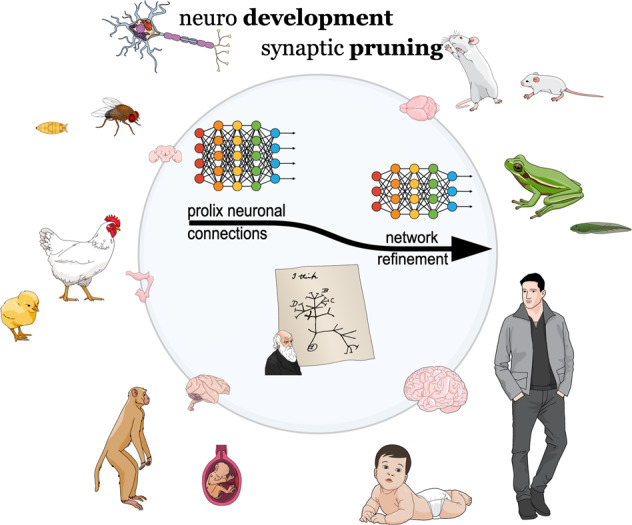
The overflowing unmatured brain - a pruning-based window of opportunities. Although the unmatured brain overflows with synaptic connections, such a developmental program goes beyond any triviality. Excessive synapses are extremely important for the brain to be able to readily fine-tune idiosyncratic connections to the demands of a rather “naïve” brain. In this way, seemingly superfluous connections make the brain prone to adapt, evolve and be molded around the behavioral repertoires that best predict continued future success. Within a concept, the time frame of promiscuous synapses could be understood as a window of opportunity in which the set of actions involving synaptic disconnections would be the tools that really makes this a good and profitable occasion. So, since the very newborn brain beginning, connections are ready to be plastically refined under the guidance of neuronal activities that mirror the nearby niche of an ever-changing environment. Furthermore, from an evolutionary perspective, pruning-based neuronal refinement of a redundant brain is likely a highly conserved mechanism as it can be found from insects to humans.

Altogether, activity-guided neuronal disconnection is part of a successful and universal developmental program, occurring naturally throughout the nervous system, as well as in different species, and timely-frame coordinated according to each network, in order to forge and fine-tune connections to then enable minute idiosyncratic adaptations that fit into individual experiences and distinct surrounding factors. Even at lower levels, be aware that the adult brain is still undergoing remodeling whereas spine lifespans vary widely. For instance, notwithstanding dendritic branches in the barrel cortex of adult mice are quite stable over weeks and close to 50% of dendritic spines may persist for at least a month, surprisingly the remaining ones are only present for a few days [41]. For the human prefrontal cortex (PFC), developmental remodeling of the brain based on the pruning of superfluous spines extends even into the third decade of life [42]. Outstanding, these latest findings from human brain enlarge neurodevelopment beyond adolescence, and such elongated phase of reorganization implies the prefrontal cortex longer vulnerable to the environment in its most labile arrangement, thus a sensitive substrate for late-onset neuropsychiatry disorders [42]. For a brief overview of the topic, see Fig. 1.

## Neuronal disconnection as a natural consequence of brain aging

**Fig. 2:**
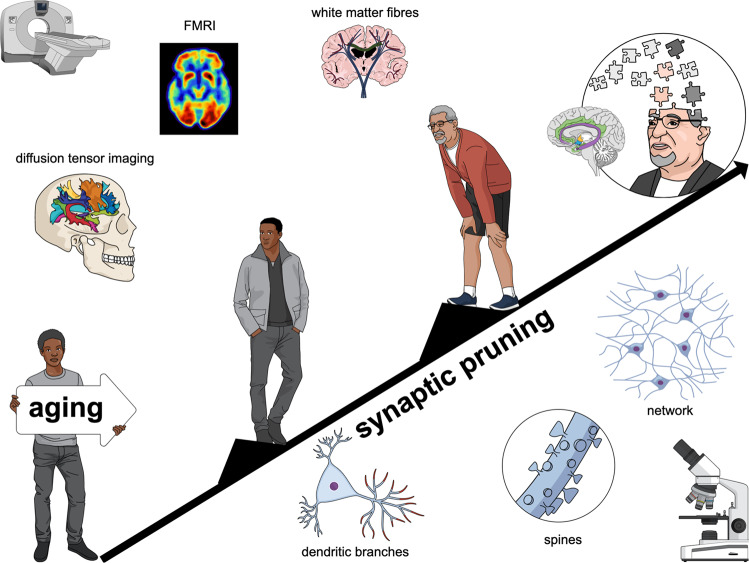
The aged and shrunken brain. After a period of developmental neural refinement based on synaptic pruning, all organisms undergo non-pathological, cumulative, and irreversible synaptic deterioration as they age. Morphological changes in aged brains may be subtle as they are region-specific and often restricted to neuronal types or dendritic branches, usually resulting in synaptic strength deficits and some sort of brain shrinkage. Although synaptic corrosion in the elderly is usually associated with some level of cognitive decline, healthy aging is expected to be unrelated to any pathological problem. However, even for healthy aging, a small cognitive decline associated with synaptic pruning can be a burden in practice as it makes the individual less able to readily adapt to the surrounding environment.

Developmental synaptic pruning related to neuronal refinement shifts over a continuum of time to a non-pathological and cumulative process of synaptic deterioration that irreversibly affects all maturing organisms, causing aging to intrinsically make the individual unable to properly adapt to the environment. Although aging is a genuine event, it is a major risk factor for the emergence of neurodegenerative and psychiatric diseases [43, 44]. Nevertheless, the occurrence of age-related cognitive decline is highly variable and is generally within the range for which aging may not yet be considered part of any neuropathology [45]. For a brief overview of the topic, see Fig. 2.

### Aging-related microstructural synaptic disconnection

Back to 1955, Brody claimed that age-linked shrinkage of the human brain was due, in part, to a decay in the number of cortical neurons [46]. Subsequent studies corroborated it by showing a decline in cortical neuron density, as well as cell loss in cortical and subcortical areas of elderly humans and non-human primates [47–49]. However, with the advancement of stereological methods new studies have identified these earlier data as confounding and likely biased by shrinkage artefacts and the mistaken inclusion of diseased samples [50, 51]. Yet, presynaptic terminal count was negatively correlated with age in individuals older than 60 years, averaging thus a 20% decrease in density of presynaptic terminals within the frontal cortex [52]. A 10% reduction per decade was also found in the total length of myelinated fibers, the main components of the white matter, adding up to 45% when the comparison was made directly between individuals aged around 20 and 80 years [53]. Similar to humans, in addition to decreased dendritic complexity, age-related changes in the non-human primate neocortex include a significant reduction in the number and density of spines [54, 55]. Further, ultrastructural inspection of white matter fiber tracts in aged rhesus monkey’s brain suggests an overall defective orchestrated driving of neuronal signals with the breakdown of the myelin sheath structure [56–58]. Indeed, such aging disconnection has been supported by independent rodent studies as, rather than loss of neurons, numerous cortical and subcortical areas show subtle, neuronal type-specific morphological changes involving the number of synaptic contacts and/or dendritic branches [59–65]. Some changes though may depend on whether the aged animal behaves cognitively normal or not [66]. Finally, even though the effects of aging are mostly drawn from the vertebrate’s brain, typically mammals, notably aged invertebrates such as insects, including fruit flies, honeybees, crickets and cockroaches also manifest a range of disconnection-like synaptic alterations [51].

### Aging-related changes in functional synaptic plasticity

While long-term potentiation (LTP) is a persistent increase in synaptic strength that favors signal transmission between neurons, its counterpart long-term depression (LTD) is a long-lasting reduction in neurotransmission efficacy [67]. Both are activity-dependent phenomena, which happen according to some pattern of electrical stimulation. In general, aging is associated with certain levels of hippocampal LTP impairment [68–73]. Interestingly, aging-related LTP deficit may depend on whether the animals show cognitive shortage [74]. As non-invasive brain stimulation methods have been increasingly useful in extending the science of LTP to humans as well [75], paired associative stimulation has shown a reduced LTP-like plasticity in the motor cortex of elderly humans [76, 77]. Alternatively to transcranial magnetic stimulation, photic tetanus induced a LTP-like visual plasticity that was not properly augmented in older humans [78]. On the other hand, older rats seem to be more sensitive to hippocampal LTD induction or LTP reversal [72, 79–81]. Strikingly, serial electron microscopy of the CA1 synapses highlighted the existence in aged mice of multi-innervated dendritic spines, which would otherwise rarely be seen [82]; while 3D reconstructions of somatic, dendritic and axonal mitochondria in DG and CA1 found niche-specific morphological alterations by comparing aged to young adult mice [83]. Both respective findings could somehow, at least partially, explain the previously described age-related changes in synaptic strength. For example, multi-innervated dendritic spines may be redundant and their assembly appears to compete for downstream signaling linked to LTP induction [82]. Besides, mitochondria play a pivotal role in Ca^2+^ buffering and therefore tune neuronal communication and plasticity, in addition to defining axonal and dendritic growth according to their location [83].

### Disturbances of cell proliferation in the aged brain

Although the brain’s cell proliferation program is extensively abolished throughout neurodevelopment, still some neuronal niches retain their proliferative precursors and thus are continually capable of producing adult-born neurons. That is particularly true for the subventricular zone of the lateral ventricles (SVZ) and the subgranular zone of the hippocampal DG (SGZ). While SVZ neuroblasts advance along the rostral migratory stream to mainly reach the olfactory bulb and differentiate into interneurons, SGZ neuroblasts shortly migrate to adjacent granular cell layer to differentiate into excitatory neurons [84]. To make this topic brief and of easier reading, below we will focus on the SGZ as it is of greater interest to humans and better studied than the SVZ.

Subpopulations of neural stem cells (NSC) in the SGZ switch from a mitotically active to a quiescent state in aged mice [85], which likely accounts for the progressive age-related impairment of proliferation, and consequently also for the disruption of either the survival, migration or differentiation of DG newborn cells [86–91]. However, both the quiescent and amplifying NSCs were actually found to continuously decrease over aging, once after exiting the quiescent state the NSC sustains a journey of asymmetric divisions to establish neuronal progenies before definitively becoming a mature astrocyte [92]. Such an interesting model to simultaneously explain aging-related phenomena involving the emergence of new astrocytes, the wane of neurogenesis and the decay of hippocampal NSCs. A prominent and progressive age-related decline in DG neurogenesis has also been found from non-human primates [93–95]. A comparison among different rodent strains and three other non-human primate species suggests that the decline in NSC proliferation, to a lesser extent also the decline in neurogenesis, actually follows absolute rather than relative age [96]. Hence, a sharp decrease in postnatal neurogenesis appears in rodents at intermediate to older phases whereas in non-human primates this happens at much earlier stages. Since a groundbreaking study revealed that the human hippocampus keeps generating neurons throughout life [97], adult neurogenesis has become a hot topic in human research. Likewise non-human primates, studies have observed in human DG that the number of neuroblasts decreases drastically over the early postnatal years, but that those remaining into adulthood still moderately decay with aging [98–100]. Making of neurogenesis actually a burning topic, some studies have contradictorily claimed that if DG neurogenesis happens during adulthood, it is nevertheless extremely rare [101, 102]. Although negative results from immunohistological *post-mortem* samples were argued to not have been optimized for detecting adult DG neurogenesis [103], even small neurogenesis levels are hypothesized to provide a reservoir of cells that would operate like a bottleneck within the reverberated hippocampal circuitry [104] as immature attributes of adult-born DG neurons appear to be long-lasting in human [105] and nonhuman primates [106] while holding a critical period of improved synaptic plasticity [107–109].

### Network-related changes in the human aged brain

By detecting and correlating activity oscillations in segregated areas, functional magnetic resonance imaging (fMRI) studies have consistently found older humans with lower connectivity between regions of the default mode network (DMN), which includes the lateral parietal cortex, precuneus, posterior cingulate (Cg) cortex, hippocampus and medial PFC - mPFC [110, 111]. The DMN maintains strong connectivity and functional organization during ongoing resting state, but decreases its functionality (intrinsic activity) under attention-demanding tasks [112]. DMN activity has been associated to several cognitive skills, in addition to assist emotional processing, recall of previous experiences and self-referential mental activities [112, 113], and reduced DMN functional connectivity in healthy aging predicts poorer performance on memory tasks and executive functions [114–116]. Resembling social networks, brain is a complex large-scale network, topologically proficient and anatomically wired to balance energetic costs, that covers subnetworks or systems/modules of highly intricate nodes - neurons or brain areas [117]. Regions of interest (ROI)- and graph theoretical-based fMRI connectome analyses broadly agree that elders at rest exhibit lower within- and higher between-modules functional connectivity than younger adults [118, 119], which suggests some level of shortage regarding the independence of brain systems. Accordingly, aging comprises less subnetworks segregation, and the lower the system specialization the worse the episodic memory scores [120]. A longitudinal study used a linear mixed modeling-based fMRI analysis to assess the complex brain networks of healthy older adults along 4 years and found that the gradual erosion of subnetworks segregation at rest correlated with aging-associated decline in cognitive performance [121]. Moreover, older adults also appear to have less modularity and less local efficiency compared to youngers [122, 123]. Modularity (number of functional modules) and local efficiency are fairly related to each other as denser local connections between the topologically closest neighbors of a node are prone to make the brain network more modular, which would favor local efficiency by enabling better adaptability of specialized information processing and its segregated transfer within reasonably autonomous dynamic brain subnetworks [117].

Age-related differences in the structural white matter connectome bring patterns of results similar to those of functional connectivity studies. For example, based on graphical theoretical indices derived from diffusion MRI tractography, older individuals were noted to have poorer anatomical organization related to global cortical connectivity (i.e. bigger network cost) and local efficiency [124]. Using a similar method, global/local network efficiency and strength of intra/inter-modular connections were observed to peak around the third decade of life to then progressively decline with aging, whereas hub (nodes with high degree of network connectivity) integration and long-range connections linearly diminished throughout aging [125]. Indeed, long-range functional connectivity density was proposed to be more susceptible to the intrinsic effects of healthy aging [126]. Tract-based spatial statistics analysis of diffusion tensor imaging data also disclosed a profile of larger extracellular volumes and lower membrane densities as a consequence of a widely disrupted white matter in a diverse set of brain structures in older compared with younger adults [127, 128]. Therefore, whether by structural or functional connectome, integrity of the brain’s network seems to undergo an overall and solid decline from midlife onwards.

## Synaptic disruption as a consequence of stress

**Fig. 3:**
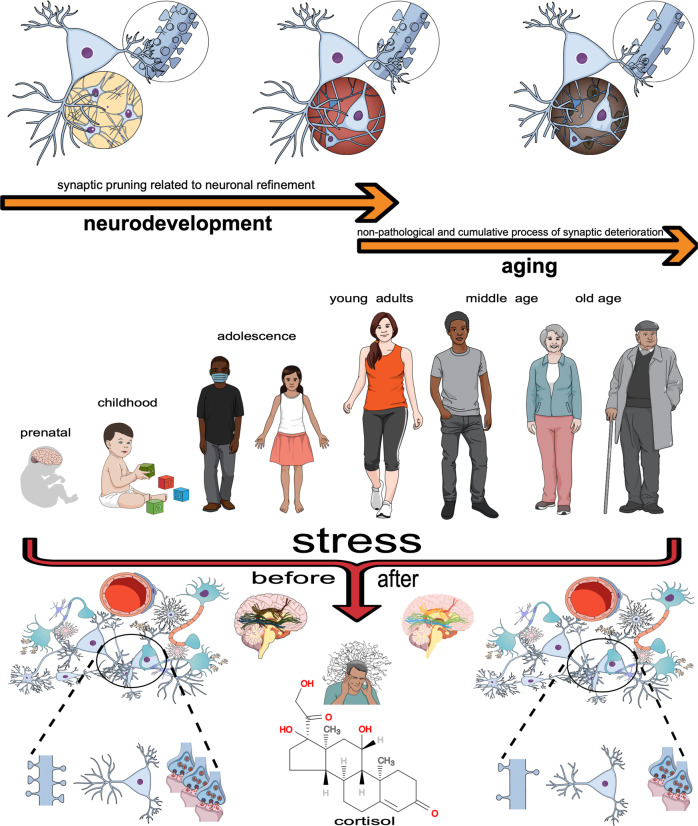
The stressed brain. As an extrinsic factor capable of altering the brain, the concept of stress is based on nothing more than our bodily ability to perceive threats, real or imagined, actual or anticipated, emotional or physical. Such a realization is then accompanied by a sense of emotional inconvenience and extensive physiological changes that should, in principle, help us orchestrate the best adaptive behavior for survival, but which may actually lead to maladaptive behavioral adjustments. Indeed, a myriad of conditions can be perceived as stressful and the extent of brain effects may vary depending on the interplay between individual resilience and how long that stress lasts. Such effects include microstructural and subsequent macrofunctional changes, both of which are usually, but not only, coupled with the triggering of synaptic disconnections. Interestingly, stress-induced brain morphofunctional changes are generally recoverable. The effects of stress can overlap with the effects of neurodevelopment and aging on the brain, as we are all susceptible to stressors throughout our lives.

Unlike to intrinsic factors driving synaptic pruning throughout neurodevelopment and aging, the ongoing art of living also includes a myriad of extrinsic factors, such as life stressors, still capable of disconnecting the brain. Stress could be conceptualized as a perception of threat, real or imagined, actual or anticipated, emotional or physical, where an individual undergoes some level of emotional discomfort and physiological changes that might lead to maladaptive behavioral adjustments. Stress responses are rather complex and dynamic, comprising a symphony of molecular and neuronal rearrangements that are coordinated at multiple levels to ideally orchestrate an optimal response to threatening challenges [129]. Thus, the stress response has originally evolved to bring the organism back to body homeostasis, protecting individuals from acute stress and maintaining their fundamental sense of well-being [130]. Additionally, in its optimal construct, the stress response may even be crucial for an individual’s optimal adaptation to the environment by triggering better experience-based homeostatic power [130]. However, prolonged exposure to stress often unbalances the stress response system towards a maladaptive homeostasis, from which several neuropsychiatric illnesses could be precipitated, including depression and post-traumatic stress disorder – PTSD [131]. Below, some stress-related brain changes. For a brief overview of the topic, see Fig. 3.

### Macrostructural changes in a stressed brain

Lifelong stress exposure may induce cumulative brain structural changes, including frontoparietal network disturbances and decreased PFC/insula gray/white matter [132–134]. Shrinkage of several cortical structures has also been reported in children who have suffered physical/sexual assault [135, 136]. Childhood stress also promotes lasting brain changes beyond youth as retrospective and prospective studies detail how early life adversities can cause the thinning of various cortical structures in adulthood [137–141]. Strikingly, personal experiences of abuse may head brain changes specifically to regions involved with that particular adverse sensory input. Accordingly, cortical structures relevant to self-awareness and self-assessment were thinner in the case of adult women emotionally abused in childhood, while early sexual abuse was mainly associated with thinning of the genital representation field lying over the primary somatosensory cortex [142]. In addition, adults exposed to parental verbal abuse during childhood showed loss of integrity in neuronal pathways involved with language development [143]. Also, the gray matter of the visual cortex is shrunken in young adults who have been sexually abused or witnessed domestic violence during childhood [144, 145]. The hippocampus of adult individuals was also found to be smaller the greater their lifetime exposure to stressful situations, including financial problems [146–148]. Additionally, the higher the level of perceived stress or the earlier the adverse experience, the lower the hippocampal volumes in adolescents [149, 150]. Also, a thorough analysis showed that adults who experienced childhood trauma had lower volumes of all hippocampal subfields [151, 152].

Therefore, a myriad of different stressors is capable of inducing neuronal disconnection, especially those carried early on life. Importantly, each type of stressor may induce specific synaptic disruption, but keep in mind that brain rearrangement goes further, as some neurocircuits are actually reinforced rather than weakened. It’s also worth noting that although we have focused primarily on data about cortical and subcortical areas, such as hippocampus, the stress-triggered changes are in fact pervasive throughout the brain [153].

### Microstructural changes in hippocampal neurons triggered by adulthood stress

Strikingly, animal studies have found that volumetric brain distortions are positively coupled to neuronal remodeling, thus convincingly demonstrating that regional shifts in spine density and dendritic arborization likely underlie gray matter changes [154, 155]. So, in accordance to previous human data, hippocampus seems to be particularly damaged after stress exposure. For example, rats exposed daily to a restraint [156, 157] or immobilization [158] stress had a decrease in the total length and branch points of the CA3 hippocampal apical dendrites. The same changes were observed in tree shrews daily subordinated to a dominant male [159]. Hippocampus is a complex structure, with a reverberant circuitry where apical dendrites of CA3 pyramidal neurons accumulate spines or thorny excrescences along their proximal segments to synapse mossy fiber inputs from DG granule neurons [160]. Hence, those previous findings probably reflect ultrastructural changes in DG-CA3 synapses, once rats chronically restraint-stressed exhibited rearrangements in vesicle clusters and mitochondrial occupancy of mossy fiber terminals [161], along with a retraction in postsynaptic thorny excrescences and reduction of their endosome-like structures [162]. Accordingly, rats chronically restraint-stressed showed extensive loss of mossy fiber contacts on CA3 thorny excrescences [163]. In fact, a thorough reorganization has been observed across all hippocampal subfields in rats under a mild but chronic and unpredictable stress regime, changes which include atrophy, decreased spine density and dendritic length [164–166]. Longitudinal MRI of rats before and after a chronic restraint stress schedule corroborated a 3% reduction in hippocampal volume [167]. Even a short resident-intruder stress paradigm promoted a prolonged decrease in dendritic length and spine density of CA1 pyramidal neurons in socially defeated rats [168]. Hippocampal cell death has also been observed short-transiently after one-day stress or long-lasting after a chronic unpredictable stress protocol [89]. Another study found increased levels of apoptosis in DG and entorhinal cortex of subordinate tree shrews who underwent to resident-intruder model [169]. Importantly, although stress can affect hippocampal neuroplasticity similarly in its most dorsal and ventral part [170], some effects may appear stronger in the ventral hippocampus [171], what would be in line with its predominant functional role in modulating neuroendocrine and emotional/motivational responses to stress [172].

### Hippocampal cell proliferation disturbances induced by adulthood stress

Although the functional role of SGZ adult-born neurons is still a matter of debate, several studies suggest that these neurons are important for neuronal pattern separation and contextual discrimination [173]. Focusing on hippocampus, hypothetically neurogenesis bias neuronal coding by avoiding proactive interferences and generalizations, making thus the brain capable of accurately updating spatiotemporal information (cognitive flexibility) to further build a proper stress response and adapt to the demands of an ever-changing environment [174]. So, it is not a surprise that stress disrupts DG neurogenesis. Accordingly, 6 weeks of rat restraint stress decreased the proliferation of DG precursor cells, attenuated the survival of adult-newborn neurons, while concurrently decreased the number of granule cells and consequently granule cell layer volume [175]. Furthermore, seven days after inescapable shocks, or 24 h after a 45-min restraint stress, rats exhibited a reduction in DG cell proliferation [176]. Indeed, escapable shocks immediately reduced DG cell proliferation while inescapable shocks induced a longer-lasting detrimental effect [177]. A 6 weeks protocol of chronic mild stress robustly attenuated DG neurogenesis by about 40% [165]. Interestingly, a day of stress affected DG cell proliferation for no longer than 24 h, however a 3-week regimen of chronic unpredictable stress induced a lasting impairment with only an incomplete recovery around the third week of no further stress [89]. Chronic mild stress also suppressed cell proliferation and reduced the total number of granule cells in the rat ventral granule cell layer [178]. Marmoset monkey’s brain is also vulnerable to stress, as a single exposure to a resident conspecific for 1 h reduced DG cell proliferation [179].

### Impairment of hippocampal synaptic strength by stress

Since there are 5000 times more synapses than neurons in the brain [180], many studies report stress largely affecting functional synaptic plasticity as well. Accordingly, the induction of LTP in adult rats, specifically from stimulation of medial perforant inputs to DG or commissural pathways to CA3, was suppressed after a 21-day schedule of restraint stress [181]. A single exposure of adult rats to an elevated platform for 30 min was able to impair hippocampal CA1 LTP in vivo and favor the appearance of a reliable LTD, which under control conditions would not occur [182]. Interestingly, prejudice in CA1 LTP was more pronounced in mature animals exposed to uncontrollable Morris water maze stress (no platform), when compared to the controllable stress group whose platform was present [183]. An impairment of CA1 and DG LTP was also observed in ex vivo hippocampal slices of grown-up animals previously stressed in a 21-day variable stress paradigm [184]. Early-postnatal stress (fragmented maternal care) also had an impact over aging rats, as middle-aged, but not young adults, presented an impairment of ex vivo CA3 LTP and a decrease in the hippocampal complexity of CA1 dendritic tree [185]. It is important to pinpoint, though, that stress will not always induce a loss in LTP, actually it may be even enhanced, or unchanged, depending on many factors such as the feature of stress and stress response phase [186]. Besides, for a long time LTP and LTD were conceived as electrophysiological phenomena predominantly implicated in brain areas whose role was associated with memory processing, such as the hippocampus. However, now it is well known that LTP and LTD are indeed widespread, from spinal cord to neocortex, and that memory storage is much more complex than previously thought and requires a large and integrative brain network [180].

### General effects of adulthood stress on cortical disconnection

The mPFC includes different subregions such as the Cg, prelimbic (PrL) and infralimbic (IL) areas, and the preclinical evidence gathered so far poses that all of these structures are largely affected by stress as well. For instance, in addition to shortening spine density, chronic daily restraint stress reduced the total length and branch numbers of the apical dendrites in Cg and PrL pyramidal neurons [187–190], an outcome that likely mirror the atrophy of terminals branches [191]. On closer examination, the same stress scheme reduced the volume, length and surface area of these apical dendritic spines, overall decreasing the density of the larger dendritic spines while increasing those of the thinner ones [192]. Seven days of daily brief restraint-stress was still capable of inducing a substantial shrinkage of the Cg apical dendrites [193]. Moreover, ten days of daily immobilization stress was sufficient to shoot down IL branch points and the overall length of apical dendrites from randomly selected or entorhinal cortex-projecting neurons [194]. Even a single exposure to the stressful forced swim [195] or elevated platform [196] arouse a retraction in apical dendrites of IL pyramidal neurons. Additionally, a chronic model of unpredictable stress led to a large volumetric shrinkage of the mPFC, as it includes all subregions, and additionally disrupted the PrL LTP acquired from high-frequency stimulation of the ventral hippocampus CA1 [197]. Excess of glucocorticoid is supposed to underlie the stress effects, at least partially, on synaptic disconnections [198]. Therefore, it is noteworthy that the brain changes triggered by corticosteroid treatment resemble those described previously for stressed animals [199].

## Importance of downward plasticity for memory

Memory is an essential cognitive function that, due to complexity, is often described apart into at least three main stages: acquisition or encoding, consolidation and retrieval. During encoding, sensory information is received by the brain, neuronal excitability is altered and synaptic plasticity starts being established. Memory is then progressively consolidated through changes in synaptic connections as it is retained as a memory trace. Finally, under predictive cues memory retrieval emerges [200, 201].

Since we are all surrounded by an ever-changing environment and constantly subject to routine bodily sensory updating, after retrieving a memory multiple destinies are still possible, such as reconsolidation, extinction and forgetting [202]. By definition, reconsolidation opens a novel temporal window of lability that allows the original memory to be modified [203, 204]. On the other hand, the extinction process occurs when a new memory trace competes with the original one [205, 206]. Forgetting, in turn, is characterized as a physiological phenomenon in which unnecessary information decays over time [207].

**Fig. 4:**
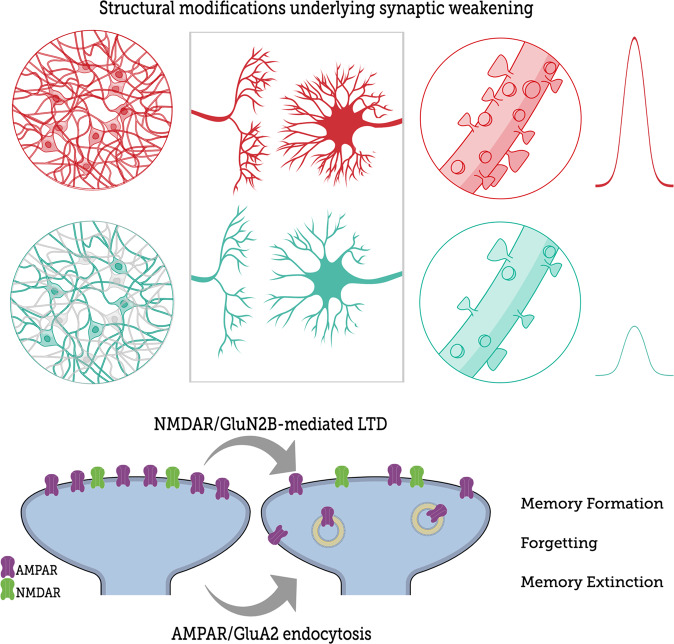
Negative plasticity is the *sine qua non* of memory. Downward structural changes such as spine removal and/or dendritic shrinkage underlie the functional weakening of synaptic strength that happens during LTD and in some specific phases of memory. Each memory phase has its particular cellular mechanism, however some of them are shared. The bottom figure abridges how AMPAR/GluA2 internalization triggered by NMDAR/GluN2B-mediated LTD is a fundamental shared cellular mechanism by which synaptic weakening happens in all these memory processes.

Here, we will focus on studies showing that downward synaptic plasticity is required for adulthood memory formation as well as for post-retrieval processes to be established. Although counterintuitive, analogous to neurodevelopment, activity-dependent synaptic disconnection is also important during the animal’s adulthood so that its brain circuits, whose neuronal coding underlies memory storage, are refined [208]. Also, suppressing neuronal ensembles associated with the foundation of a previous memory may favor subsequent learning/memory by reducing any potential interference [209]. For a brief overview of the topic, see Fig. 4.

### Impairment of synaptic strength for learning and memory

Classical studies correlate learning and memory with increased synaptic efficacy [210]. However, there is currently no doubt that decrement in synaptic efficacy may also support memory formation [211–214]. Accordingly, spatial learning triggered endogenous LTD [215, 216]. While LTD and LTP share some molecular mechanisms that are required for memory formation, e.g. activation of N-methyl-D-aspartate receptors (NMDARs), unlike LTP, LTD requires endocytosis of α-amino-3-hydroxy-5-methyl-4-isoxazole propionic acid receptors (AMPARs), which are generally found in postsynaptic density [211, 217, 218]. Hence, blocking the GluN2B subunit of NMDARs, or AMPAR endocytosis, disrupted both LTD- and hippocampus-dependent learning and memory [219–222]. Additionally, it was demonstrated that LTD mediated by GluA2 (AMPAR subunit) endocytosis in the apical inputs to CA1 neurons is crucial for the establishment of place fields during spatial learning [223]. At the circuitry level, it was shown that new experiences activate the locus coeruleus to release noradrenaline into the hippocampus, mediating thus spatial learning through LTD along Schaffer collaterals-CA1 synapses [224]. Therefore, it is clear that synaptic weakening and its underlying cellular mechanisms are essential for shaping hippocampus-dependent spatial memories.

### Weakening of synaptic strength during memory extinction and forgetting

Extinction and forgetting may involve functional and structural reorganization of synapses that were potentiated throughout the original learning [207, 225]. First, be aware that the neurobiological mechanism underpinning the reduction in synaptic strength, in addition to LTD, might also imply depotentiation (induction of synaptic depression following LTP) and LTP decay [226]. Recent studies, in fact, have shown that the blockade of NMDARs in the hippocampus prevents forgetting [227] and also the LTP decay [228, 229], indicating that forgetting actually takes place as an active rather than a passive process. In addition, NMDAR-mediated calcium entry activates calcineurin [229] and synaptotagamin-3 [230], both of which ultimately lead to the removal of GluA2-AMPARs from hippocampal synapses and cause a reduction in synaptic strength [230, 231], then favoring forgetting. Likewise, blocking synaptic removal of GluA2-AMPARs prevented depotentiation [231]. Comparably to forgetting, GluA2-AMPAR endocytosis has also been implied on memory extinction [232]. Accordingly, extinction reduced the surface expression of AMPAR subunits to pre-conditioning levels whereas depotentiated the conditioning-induced synaptic potentiation from the internal capsule to the lateral amygdala in a NMDAR-dependent manner [233]. The neuronal response to fear conditioned tone was indeed shown to return to baseline levels after extinction, thus indicating that the synaptic connection from auditory sensory input to the lateral amygdala is somehow reset by extinction [234]. Extinction memory was also shown to suppress the reactivation of contextual fear engram cells while activating another distinct ensemble in the hippocampus [235]. At last, synaptic depression of basolateral amygdala (BLA) inputs to PrL and IL mPFC improved extinction memory as it led to a more efficient reduction in fear expression [236].

Although we have focused on describing how the decreased synaptic strength/activity of a previously fear consolidated memory might be a crucial mechanism for extinction and forgetting, it is worth mentioning that all memory phases are regulated by a complex and integrated brain circuit. Part of which might involve different input/output or modulatory pathways that would alternatively be enhanced [237–239].

### Structural modifications underlying synaptic weakening and memory

Plenty of studies have associated shrinkage and elimination of dendritic spines with synaptic LTD [240–242]. In detail, an optically-induced LTD provoked a homogeneous lowering of hippocampal synaptic function, followed over days by synaptic refinement, as synapses most likely to release neurotransmitters regained their function over time whereas low-probability ones were removed [208]. On the other hand, repetitive induction of chemical LTD on consecutive days resulted in substantial retraction of mushroom spines, whose subtype is considered the most stable [243]. Additionally, mutant animals lacking the GluN2B subunit of NMDAR, in addition to deficits in learning and LTD expression, had an expressive reduction on dendritic spine density in CA1 neurons [219]. When it comes to memory-related dendritic shrinkage, structural alterations were supposed to engender the synaptic refinement vital for stablishing memory once fear conditioning induced a reduction in spine density specifically in hippocampal neurons that were active during learning, but not in those inactive [244]. Besides, fear conditioning was found to induce spine elimination also in other brain regions such as motor [245] and frontal association cortex [246]. The neurobiology of extinction memory has also been grounded on a complex structural remodeling that involves several brain areas. Although it is widely accepted that extinction has its foundations built on a new memory, following studies have implied that the original conditioned trace may still be affected by extinction as, in addition to the increase in number, all the structural spine changes of the anterior Cg, IL, BLA and auditory cortex were found reset back to preconditioning levels by extinction [247–249]. Overall, spine or dendritic removal/shrinkage underlies functional synaptic weakening during some particular phases of memory, such a scenario that gets more complex when entangled with upward neuroplasticity.

## The downward flow of homeostatic plasticity

The intracellular measurement of excitatory and inhibitory synaptic input ratios establishes the key principle of excitatory and inhibitory (E/I) synaptic balance, a concept which helps to better understand how the neuronal firing rate is sustained within a physiological range and under a neurocircuitry perspective to maintain accurate and reliable any transmission of information. E/I ratios are kept stable over time despite fluctuating external interferences, and the mechanisms underlying its maintenance are diverse and intricate [250]. Tight coupling with a counterweight is thought to regulate how quickly and accurately neurons can respond to a stimulus, once it would function as gain and selectivity mechanisms so that it might favor output refinement while expanding the operational range necessary to drive neuronal activity [251].

Within the perspective of a single neuron the firing rate is homeostatically regulated by neuronal intrinsic excitability via voltage-dependent inward and outward currents [252], as such adjustment dictates how easily neurons will reach spike threshold [253]. Furthermore, postsynaptic activity dynamically adjusts the induction of plasticity through a sliding threshold between potentiation and depression so that it also happens to modulate subsequent plasticity - i.e., metaplasticity [254, 255]. When past activity is low, the synaptic threshold slides down and favors LTP induction, conversely higher overall activity slides the threshold up and favors LTD induction [256]. Additionally, it is assumed that homeostatic adjustments in synaptic strength are accompanied by changes in the accumulation of postsynaptic receptors such as NMDAR and AMPAR, characterizing synaptic scaling [257, 258]. All these changes can occur locally, at synaptic sites, or globally throughout the entire dendritic arborization. An important hallmark of *synaptic scaling* is that the number of synaptic receptors is modified following a multiplicative scaling factor so that preserves the relative differences between synaptic weights and properly conserves the information stored [259–261].

At a spine structural level, competitive interactions between spines are expected to maintain total excitatory inputs constant, within a dynamic range, as increased spine density was followed by decreased spine volume and individual synaptic response [262] and theta burst stimulation-induced LTP increased spine size while decreased overall spine density [263]. Indeed input-specific synaptic potentiation induced with high-frequency glutamatergic uncaging led to structural growth of the local dendritic spine of hippocampal CA1 pyramidal neurons, but shrank and weakened nearby unstimulated spines [264]. The same pattern of such heterosynaptic shrinkage of inactive and adjacent spines was witnessed in the principal neurons of the basolateral amygdala [265]. Accordingly, efficacy of spontaneous transmission in both cortical and hippocampal individual synapses was regulated by the extent of nearby synaptic co-activity [266]. Interestingly, activity-driven shrinkage of neighboring spines was limited to 10 µm radius inter-spine distance and the closest ones had the greatest shrinkage [267]. It is thought that heterosynaptic spine elimination could contribute to the compartmentalization of dendritic segments by clustering synaptic inputs, thus favoring the dendritic branch as a fundamental functional unit able of performing local computations and memory storage [268, 269].

**Fig. 5:**
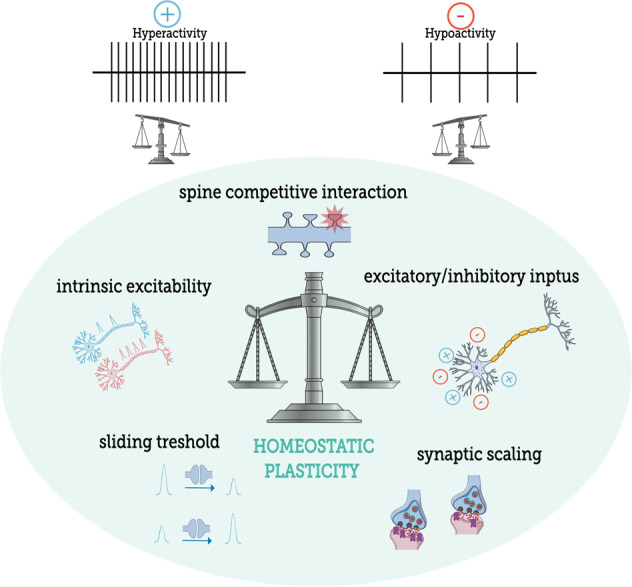
Homeostatic mechanisms controlling fluctuations on brain activity. Homeostatic plasticity is a global mechanism that comprises several forms of controlling brain activity within adequate physiological levels. Hyperactivity mobilizes downward homeostatic mechanisms that act by braking down and constraining neuronal activity over a physiological range. On another hand, a counteracting upward homeostatic force is recruited following hypoactivity. Regular cognitive process such as memory are tempered by homeostatic plasticity. The same regulation happens when individuals have to deal with stressful challenges. Since neurodevelopment, homeostatic mechanisms are observed and expressed in particular ways depending on the brain area, cell-type and age investigated. Also, homeostatic dysregulation may explain some of the aging-related phenomena such as hyperexcitability.

From the standpoint of an entangled brain, this complex set of homeostatic regulatory mechanisms is important for modulating synaptic weights and neuronal activity in order to maintain neuronal network homeostasis at both spatial and temporal scales [259]. Although data on homeostatic plasticity are scarce when not focused to its own neurophysiological mechanisms, to make a better sense of this topic as an extension of our other topics we next describe some studies in which homeostatic plasticity has someway been involved with some aspect related to neurodevelopment, aging, stress and memory. For a brief overview of the topic, see Fig. 5.

### Neurodevelopment - homeostatic plasticity entailed to low brain activity

The extent which and location where homeostatic control takes place may be impacted by circuit maturation throughout neural development as in the visual cortex it is triggered by visual experience. For example, at postnatal day 16 to 21, layers 4 and 6 are under synaptic scaling regulation [270], while on later ages scaling switches to layers 2/3 [271, 272]. Disturbances of activity in the developing network may also be restored to normal levels with inhibitory and/or excitatory adjustments. Consistently, the density of inhibitory synapses decreased when a glutamate receptor antagonist was applied to the organotypic culture of the developing hippocampus [273]. In addition, homeostatic plasticity may involve specific cell-types as developing hippocampal granule cells exhibited reduced inhibitory input from parvalbumin-positive basket interneurons when excitatory drives were absent [274]. Pyramidal neurons in layer 4 of the visual cortex dynamically adjusted excitatory and inhibitory inputs, respectively, up and down to compensate for the reduction in sensory cues [275].

### Homeostatic plasticity involved with high brain activity of adult animals

#### Memory

Based on the central idea that learning induces an increase in neuronal excitability a study observed that the compensatory reduction in hippocampal neuronal excitability, induced with the delivery of a high-frequency spike train specifically to fear engram cells, facilitated memory extinction while decreased the density of dendritic spines and increased the number of inhibitory synapses [276]. Contrary to the idea that neurons with higher excitability are more likely to be recruited for learning while learning itself would increase neuronal excitability [277–279], learning-induced hyperexcitability actually happened to rapidly trigger a counteracting force sufficient to decrease neuronal excitability [280]. However, in this case such homeostatic plasticity was suggested to be critical for consolidating memories without interference. Another study found that synaptic refinement induced by homeostatic downscaling sculpted an accurate associative taste memory after taste aversion conditioning initially induced a generalized response as synapses in the gustatory cortex were potentiated [281].

#### Stress

Homeostatic control might act as a protective physiological mechanism that supports appropriate coping strategy during stressful circumstances by maintaining the dynamics of behavioral and physiological adjustments within an optimal range through a precise E/I neuronal balance [282]. On the other hand, failure of homeostatic controls during stress might favor depression and anxiety-like behaviors [283]. Interesting, a study has demonstrated that resilient mice expressed an up-regulated hyperpolarization-activated current (I_h_) in the dopaminergic neurons of the ventral tegmental area (VTA) after chronic stress. This I_h_ current was higher than the upregulation usually observed in depressed mice and it drove an excitatory force that was countered by an increase in K^+^ channel currents, a cell-specific compensatory mechanism that reduced VTA DA excitability to control levels [284].

#### Aging

Accumulating evidence demonstrates that neuronal hyperexcitability and hyperexcitable networks are hallmarks of normal [285, 286] and pathological aging [287]. Also, hyperexcitability in different brain regions as the hippocampus [285, 286, 288] and cortex [289] is associated with cognitive deficits [290, 291]. Many factors may account for hyperexcitability, including dyshomeostasis in Ca^2+^ [287, 291], alterations of GABAergic [292, 293] or glutamatergic [286] circuitry with effects on the E/I balance, as well as modifications on intrinsic excitability [288, 294]. Hypothetically, these age-associated changes could underlie failures on homeostatic control of excitability and influence neuronal firing rate [295]. Numerous proteins involved in AMPAR stabilization and AMPAR-mediated signaling cascades are down- or upregulate in the whole aging brain [296, 297], a potential mechanism for synaptic scaling and homeostatic control failures.

## Concluding remarks

Since the brain was found to be somehow plastic, scientists urge for boosting this power up. Misleadingly, this often means increasing neuronal connections, once disconnections have been indiscriminately linked to all sorts of brain disorders. So, it is not uncommon to find “neuroplasticity impairment” in the scientific literature when it comes to data somehow related to synaptic disconnections - for an interesting review about molecular mechanisms of dendritic spine elimination, please see [242]. Although brain plasticity may have different primary minimalist meanings for neurodevelopment (redundancy and neuronal refinement), aging (synaptic deterioration and higher risk of neuronal disease), stress (adaptation or higher risk of psychiatric illness), and memory (learning and adaptability), it takes place on an interwoven continuum where neuroplasticity is indeed beyond the boundaries of any biological morality. Just as Bob Dylan criticizes how prior generation deals with social evolution and ends up saying “the times they are a-changin”, we propose a rationale for a paradigm shift that will hopefully shed some light on how synaptic disconnections fit within the concept of neuroplasticity.

Once aware of the broader picture regarding the role of brain flexibility, we should primarily speak of neuroplasticity as a balance between what we have named “upward” and “downward” neuroplasticity. Just common binary words to describe each path in which neuroplasticity could move along a direction vector. So, whatever route neuroplasticity takes, including whatsoever organically leads to synaptic disconnection or weakening, it does as a consequence of the brain’s ability to change itself. One could still say that once a synapse is gone, the brain would end up with lower levels of neuronal matter for the next move of neuroplasticity to take place. Indeed, neurodegenerative disorders make the brain less flexible as brain mass losses become massive over the course of the disease. So, for these cases it is unavoidable to think of an impairment of neuroplasticity. The same could be said about brain injury or stroke brain damage. However, under physiological conditions such as neurodevelopment, healthy aging, stress coping, memory and learning, synaptic turnover is part of a contingency plan to optimize the flow of neural information on demand. So, considering how complex the brain and its entangled neurocircuitry are, it is not worth compartmentalizing it to refine neuroplasticity concepts based on microniches as any change might be directly or indirectly counteracted at the cellular or neurocircuitry level by a completely opposite direction of neuroplasticity.

While we attempt to reduce the bias related to any moral judgment that could be even loosely associated with disconnection of synapses, we are aware that this approach is just the beginning as a paradigm shift has much more to deal with than handling sub-concepts. Indeed, any bias depends on the guided experience of a personal life, and therefore the concept of “downward” or “upward” neuroplasticity might still have some negative or positive connotation. We then want to make it as clear as possible that the idea of using such sub-concepts comes from the neutral view of direction vectors, an approach that should at least help to minimize any personal moral bias towards neuroplasticity.

As the island of knowledge grows larger, so does its horizon. The same happens when we include the “upward” and “downward” concepts to neuroplasticity. So now more than ever is a great opportunity to update the meaning of neuroplasticity, as our current knowledge that plasticity is an intrinsic and bidirectional property of neurons somewhat blurs the difference between “neuroplasticity” and “neurophysiology”. Although it sounds more of an epistemological issue, we propose that neuroplasticity is the ability of the brain to change itself in such a way that any gain or loss in brain function should be bigger or lower than the sum of its lost or gained parts. So, brain reorganization itself would not suffice to determine neuroplasticity without an amplified gain or loss of brain function, provided that the initial state of the brain is maintained and there is no change in the specific function of one region that is sufficient to reach and modify other areas of the brain. Such an updated concept would fit better with the new findings that place any single part of the brain directly or indirectly connected to any other single part of the brain, making even a small change likely to influence the entire brain in some way.

**Fig. 6:**
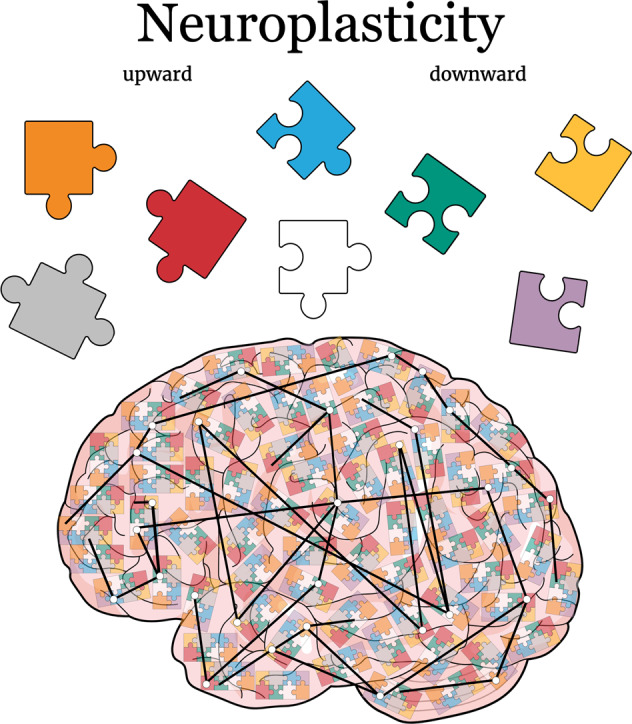
Downward neuroplasticity within the larger and complementary perspective of an entangled brain. The main goal of this review is to work on the idea of how neuroplasticity, in all its complexity, should be conceptually understood as a balance between what we have called here as “upward and downward neuroplasticity”. Developing brains, and partially also the adult ones, are flexible, moldable, and as it is, any capability for decreasing the density levels or structural complexity of spines and/or dendrites should be considered part of the neuroplasticity program, rather than being a deficiency of it. Thus, comparable to how puzzles fit together, upward and downward neuroplasticity work to complement each other so that the brain would eventually be able to reshape its connections by neuronal tuning to optimize network’s efficiency under certain demands. Within a broader landscape as seen with assembled puzzles, although neuroplasticity happens at first glance from the microscale changes of spines and dendrites according to a neuronal perspective, its consequences expand toward a macroscale outlook where individual orchestrated changes integrate into the account of different neuronal populations and neurocircuits. So, any cause or consequence neuroplastic change from an entangled brain, whether up or down, may be directly or indirectly connected to at least some other part of the brain that could still show a completely opposite direction of neuroplasticity. The orange, red and gray puzzles represent different populations of neurons that in the sum of the events globally present upward plasticity, while the green, yellow and purple puzzles represent different populations of neurons that in the sum of the events present global downward plasticity. Meanwhile, the blue and white puzzles represent neurons with a balanced number of events that represent both upward and downward neuroplasticity. Their connections should maintain the brain largescale of neuroplasticity at zero sum when considering downward and upward neuroplasticity within the topography of neuronal matter.

Therefore, upward and downward plasticity should at first be understood as complementary to each other, including in cases of psychiatric disorders. Both neuroplasticities co-exist, certainly interact with each other and thus one has nothing more especial than the other. Hence, it is important to give downward neuroplasticity as much attention as upward neuroplasticity given that brain flexibility would not be complete without one or the other. For good or bad, downward modifications are part of the neuroplasticity program, rather than being a deficiency of it. For a brief overview of the conclusion, please see Fig. 6.
